# Peripersonal and reaching space differ: Evidence from their spatial extent and multisensory facilitation pattern

**DOI:** 10.3758/s13423-021-01942-9

**Published:** 2021-06-22

**Authors:** A. Zanini, I. Patané, E. Blini, R. Salemme, E. Koun, A. Farnè, C. Brozzoli

**Affiliations:** 1 ImpAct Team, Lyon Neuroscience Research Centre, INSERM U1028, CNRS UMR5292, Lyon, France; 2 University Claude Bernard Lyon I, Université de Lyon, Lyon, France; 3 Department of General Psychology, University of Padova, Padova, Italy; 4 Hospices Civils de Lyon, Neuro-immersion – Mouvement et Handicap, Lyon, France; 5 Center for Mind/Brain Sciences, University of Trento, Rovereto, Italy; 6 Department of Neurobiology, Care Sciences and Society, Aging Research Center, Karolinska Institutet, Stockholm, Sweden

**Keywords:** Peripersonal space, Hand-centered space, Reaching space, Multisensory, Perception

## Abstract

Peripersonal space (PPS) is a multisensory representation of the space near body parts facilitating interactions with the close environment. Studies on non-human and human primates agree in showing that PPS is a body part-centered representation that guides actions. Because of these characteristics, growing confusion surrounds peripersonal and arm-reaching space (ARS), that is the space one’s arm can reach. Despite neuroanatomical evidence favoring their distinction, no study has contrasted directly their respective extent and behavioral features. Here, in five experiments (*N* = 140) we found that PPS differs from ARS, as evidenced both by participants’ spatial and temporal performance and by its modeling. We mapped PPS and ARS using both their respective gold standard tasks and a novel multisensory facilitation paradigm. Results show that: (1) PPS is smaller than ARS; (2) multivariate analyses of spatial patterns of multisensory facilitation predict participants’ hand locations within ARS; and (3) the multisensory facilitation map shifts isomorphically following hand positions, revealing hand-centered coding of PPS, therefore pointing to a functional similarity to the receptive fields of monkeys’ multisensory neurons. A control experiment further corroborated these results and additionally ruled out the orienting of attention as the driving mechanism for the increased multisensory facilitation near the hand. In sharp contrast, ARS mapping results in a larger spatial extent, with undistinguishable patterns across hand positions, cross-validating the conclusion that PPS and ARS are distinct spatial representations. These findings show a need for refinement of theoretical models of PPS, which is relevant to constructs as diverse as self-representation, social interpersonal distance, and motor control.

**Supplementary Information:**

The online version contains supplementary material available at 10.3758/s13423-021-01942-9.

## Introduction

Seminal studies described multisensory neurons in primates’ fronto-parietal regions coding for the space surrounding the body, termed *peripersonal space* (PPS) (Colby et al., 1993;Graziano & Gross, 1993 ; Rizzolatti et al., 1981a, 1981b). These neurons display visual receptive fields anchored to tactile ones and protruding over a limited area (~5 to 30 cm) from specific body parts (e.g., the hand) (Graziano & Gross, 1993; Rizzolatti et al., 1981a, 1981b). Neuroimaging results in humans are in line with these findings: ventral and anterior intraparietal sulcus, ventral and dorsal premotor cortices and putamen integrate visual, tactile and proprioceptive signals, allowing for a body part-centered representation of space (Brozzoli et al., 2011, 2012). Behaviorally, visual stimuli modulate responses to touches of the hand more strongly when presented near compared to far from it (Farnè et al., 2005; Làdavas & Farnè, 2004; Serino et al., 2015; Spence et al., 2004), a mechanism proposed to subserve both defensive (de Haan et al., 2016; Graziano & Cooke, 2006) and acquisitive aims (Brozzoli et al., 2009, 2010; Brozzoli et al., 2014; De Vignemont & Iannetti, 2014; Patané et al., 2019).

As a multisensory interface guiding interactions with the environment, PPS shares some characteristics with the arm-reaching space (ARS), the space reachable by extending the arm without moving the trunk (Coello et al., 2008). In humans, ARS tasks typically require judging the reachability of a stimulus (Carello et al., 1989; Coello & Iwanow, 2006). Despite their anatomo-functional differences (Desmurget et al., 1999; Filimon, 2010; Lara et al., 2018; Pitzalis et al., 2013), some research on human PPS diverged from the original electrophysiological findings and combined ARS and PPS (Coello et al., 2008; Iachini et al., 2014; Vieira et al., 2020). However, multisensory stimuli within ARS and close to the hand activate neural areas typically associated with PPS, whereas the same stimuli within ARS, but far from the hand, do not (Brozzoli et al., 2012; Graziano et al., 1994). To date, no empirical evidence exists to distinguish these spatial representations. The consequences of this conflation on spatial models of multisensory facilitation have to date been neglected, despite the crucial role it plays in sensorimotor control (Makin et al., 2017; Suminski et al., 2009, 2010) and the study of the bodily self (Blanke et al., 2015; Makin et al., 2008).

Here we leveraged empirical outcomes to disentangle two alternative theoretical models, hypothesizing that PPS and ARS are either identical or distinct spatial representations. To ensure fair comparative bases for this purpose, and to allow making clear alternative predictions, we set two pre-requisites: (1) not to oppose PPS and ARS in the context of different functions, and (2) to test both spaces with reference to the same body part. Thus, in Experiment 1 we used a tactile detection task and computed multisensory (visuo-tactile) facilitation, a typical proxy of PPS extent. In Experiment 2 we used a reachability judgment task and computed the point of subjective equality (PSE), a typical estimate of the ARS extent (Bourgeois & Coello, 2012). As visual and tactile stimuli were harmless and semantically neutral, our tasks were devoid of any defensive or social function. In addition, both PPS and ARS tasks were applied in reference to the hand, as PPS has been shown to be hand-centered (di Pellegrino et al., 1997) and what we can reach (ARS) is defined by how far our hand can reach (Coello & Iwanow, 2006), thus fulfilling the criteria for a fair comparison. Two additional experiments manipulated hand vision (visible or not) and position (close or distant), to progressively equate the reachability task to the multisensory conditions of Experiment 1.

Following this rationale, if PPS and ARS are equal, we should observe similar spatial extents from multisensory facilitation and reachability estimates. In addition, we should observe facilitation from all visual stimuli falling within ARS independently of hand position. Conversely, we should measure different spatial extents and observe multisensory facilitation only for stimuli near the hand, as a function of its position, resulting in specific and distinguishable spatial patterns of multisensory facilitation.

## Experiment 1

### Methods

#### Participants

We calculated our sample size with G*Power 3.1.9.2, setting the 10*2 (V-Position*Hand Position) within-interaction for a RM ANOVA hypothesizing a power of 0.85, an α = 0.05 and a correlation of 0.5 between the measures. We assumed that the visuo-tactile effect size might be greater than the audio-tactile one (small, corresponding to Cohen's d=0.2 so to f = 0.1) reported by Holmes and colleagues (Holmes et al., 2020). We thus considered a medium-low effect size (f = 0.20) and we needed to recruit at least 23 participants per study. All participants were right-handed, as evaluated via the Edinburgh Handedness Test (mean score 82%). Twenty-seven subjects (13 females; mean age = 26.12 years, range = 20–34; mean arm length = 79.41 ± 5.83 cm, measured from the acromion to the tip of the right middle finger) participated in Experiment 1.

All participants reported normal or corrected-to-normal vision, normal tactile sensitivity, and no history of psychiatric disorders. They gave their informed consent before taking part in the study, which was approved by the local ethics committee (Comité d’Evaluation de l’Ethique de l’Inserm, n° 17-425, IRB00003888, IORG0003254, FWA00005831) and was carried out in accordance with the Declaration of Helsinki. Participants were paid 15 € each.

#### Stimuli and apparatus

**Fig. 1:**
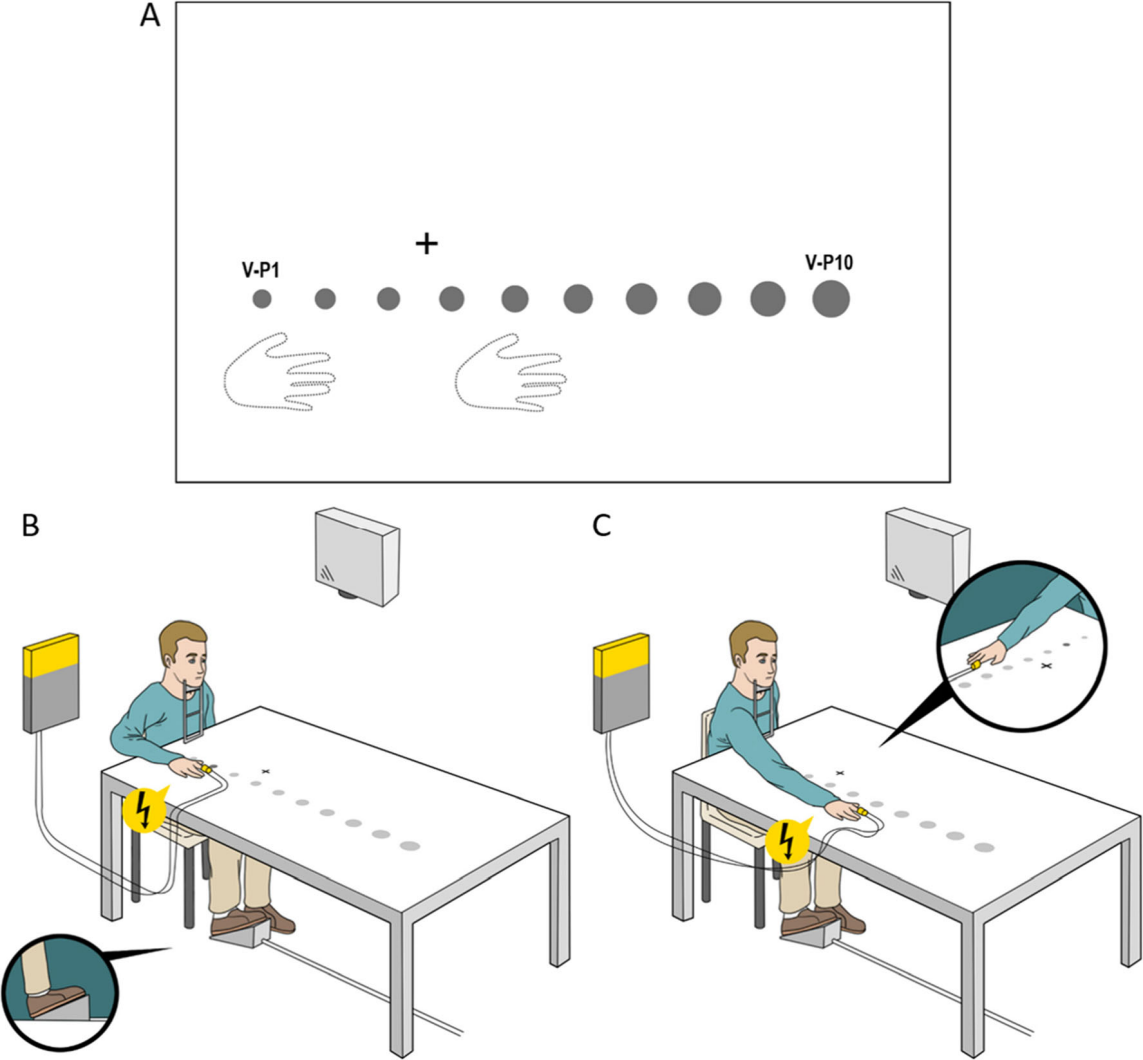
Experimental setup across experiments. **a** Positions of right hand, fixation cross, and visual stimuli. **b** and **c** The close hand (**b**) and the distant hand condition (**c**). In both experiments, the visual stimuli (here displayed as gray circles) were projected one at a time, in one of the ten possible positions (from V-P1 to V-P10), corrected for retinal size (**a–c**). Tactile and visual stimuli were presented alone (unisensory) or coupled synchronously with each other (multisensory). Globally, we adopted two conditions of unisensory stimulation (only tactile or visual stimulation) and a multisensory condition (visuo-tactile stimulation). To these, we added catch trials (nor visual nor tactile stimuli presented) to monitor participant’s compliance

*Visual stimuli* were identical for both the experiments. We used a projector (Panasonic PT-LM1E_C) to present a two-dimensional (2D) gray circle (RGB = 32, 32, 32) in one of ten positions, ranging from near to far from the body. The diameter of the gray circle was corrected for retinal size using the formula:

$$ \frac{3{cm}^{\ast}\left(57 cm+x\right)}{57 cm} $$

where 3 cm is the diameter of the circle, 57 cm is the distance from the eye at which 1° of the visual field roughly corresponds to 1 cm, and *x* is the distance of the center of the stimulus from the point at 57 cm. Visual stimulus duration was 500 ms. The fixation cross (2.5 cm) was projected along the body’s sagittal axis (see Fig. 1). The ten positions were calibrated such that the sixth one corresponded to the objective limit of reachability for each participant. We ensured this before the experiment: participants stayed with eyes closed, their head on a chinrest (30 cm high), and placed their right hand as far as possible on the table. Starting from the sixth position, four positions were computed beyond the reachable limit and five closer to the participant’s body, 8 cm rightward with respect to the body’s sagittal axis. Positions, uniformly separated by 9 cm, spanned along 90 cm of space and were labelled V-*P1* to V-*P10,* from the closest to the farthest (see Fig. 1).

*Tactile stimuli* were brief electrocutaneous stimulations (100 μs, 400 mV) delivered to the right index finger via a constant current stimulator (DS7A, DigiTimer, UK) through a pair of disposable electrodes (1.5*1.9 cm, Neuroline, Ambu, Denmark). Their intensity was determined through an ascending and a descending staircase procedure, incrementing and decrementing, respectively, the intensity of the stimulation to find the minimum intensity at which the participant could detect 100% of the touches over ten consecutive stimulations. Intensity was further increased by 10% before the first and third experimental block.

#### Design and procedure

Participants performed a speeded tactile detection task. Tactile stimulation of their right index finger could be delivered alone or synchronous to a visual one, in one of the ten positions (see Fig. 1). Participants rested with their head on the chinrest and eyes on the fixation cross. Their right hand was placed on the table 16 cm rightward from the body’s sagittal axis, with the tip of the middle finger corresponding to V-P2 (hereafter close hand) or V-P6 (hereafter distant hand), in different blocks counterbalanced across participants (116 randomized trials per block): two blocks with the close hand and two with the distant hand. Considering the distance between the positions of visual stimulation, the hand in the distant position covers positions V-4 (wrist), V-5, and V-P6 (tip of the middle finger), and the hand in the close position is flanked by the positions V-P1 and V-P2 (see Fig. 1). Each hand condition included 16 visuo-tactile (VT) stimulations per position and 16 unimodal tactile trials (T trials). To ensure compliance with task instructions, there were also four unimodal visual trials per position (V trials) and 16 trials with no stimulation (N trials). Participants had to respond to the tactile stimulus as fast as possible by pressing a pedal with their right foot. The total duration of the experiment was about 45 min.

#### Analyses

Both the experiments adopted a within-subject design. When necessary, Greenhouse-Geisser sphericity correction was applied. The first analyses focused on the accuracy of the performance. Four participants performed poorly (>2 SD from mean) and were excluded from further analyses.

To have a direct index of the proportion of multisensory facilitation over the unimodal tactile condition, we calculated the Multisensory Gain (MG):

$$ MG=\frac{T_M- VT}{T_M} $$

T_M_ was the average reaction time (RT) for unimodal tactile stimuli, and VT was the raw RT for a multisensory visuo-tactile stimulus. Larger MG values correspond to greater facilitation (namely, larger benefits for VT compared to T conditions). This measure is more rigorous than an absolute delta, as it allows correction of the RTs considering the subject-specific speed for each visual position and for each position of the hand (analyses on the delta RT are also reported in the Appendix – Experiment 1). Computing MG values per hand and stimulus position, we obtained two vectors of 10 MG values (from V-P1 to V-P10) for each participant: one for the close hand and one for the distant hand. We applied a multivariate SVM approach (Vapnick, 1995) to test whether a data-driven classifier could reliably predict the position of the hand from the spatial pattern of MG. The SVM was trained on (N – 1) participants (leave-one-out strategy) and tested on the two vectors excluded from training, using a linear kernel. Overall accuracy was calculated as the sum of the correct predictions for both hand positions divided by the total number of predictions.

To map multisensory facilitation more locally, we compared Bonferroni-corrected MG values for each position against zero and performed a *Hand* (close vs. distant)*Position (V-P1 to V-P10) within-subject ANOVA.

To compare the shape of these multisensory facilitation maps, we first tested which function better fit the spatial pattern and, second, we cross-correlated them to test their shapes for isomorphism. MG values were fitted to sigmoidal and normal curves, limited to two parameters. Table 1 reports formulas for curve fitting (Curve Fitting toolbox) with MATLAB (version R2016b, MathWorks, Natick, MA, USA). Similar to previous work (Canzoneri et al., 2012; Serino et al., 2015), we considered a good sigmoidal fit when data fitted a descending slope, indicating a facilitation close to the body that fades away with increasing distances.

**Table 1:** Formulas adopted to fit the curves for the multisensory gain values in Experiment 1. X represents one of the ten experimental positions (from V-P1 to V-P10). We used the same formulas to fit the sigmoidal and normal curves to reachability judgments in Experiment 2

Sigmoidal	Normal
$\frac{100}{1+{e}^{-a\left(X-b\right)}}$	$100\ast {e}^{{\left(\frac{X-a}{b}\right)}^2}$

Next, we performed a cross-correlation analysis on MG values to evaluate the isomorphism of the facilitation curve for both hand positions. Our prediction was that shifting the close hand pattern of facilitation distally (i.e., towards the distant-hand position), should bring higher correlations due to the overlap of the curves. We correlated the pattern of averaged MG values for all reachable stimuli (V-P1 to V-P6) in the close hand condition, with that of six averaged MG values observed in the distant hand condition. The correlation was then tested for four incremental position shifts (distally, one per position), up to the last shift, where we correlated the V-P1 to V-P6 pattern for the close hand with the V-P5 to V-P10 pattern of the distant hand.

### Results

We tested the effect of VT stimulation over ten uniformly spaced positions, to obtain a fine-grained map of patterns of multisensory facilitation (validated in a pilot study)*.* Participants performed accurately (90% hits, < 2% false alarms). First, the multivariate classifier was able to predict the two positions of the hand with an accuracy of 0.72 (33/46 correct classifications), with no bias for one hand position over the other (17/23 and 16/23 for the close and distant hands, respectively). This accuracy was significantly higher than chance (one-tailed binomial test *p* = 0.002). Hence, different patterns of multisensory facilitation were associated with different hand positions within the ARS.

**Fig. 2:**
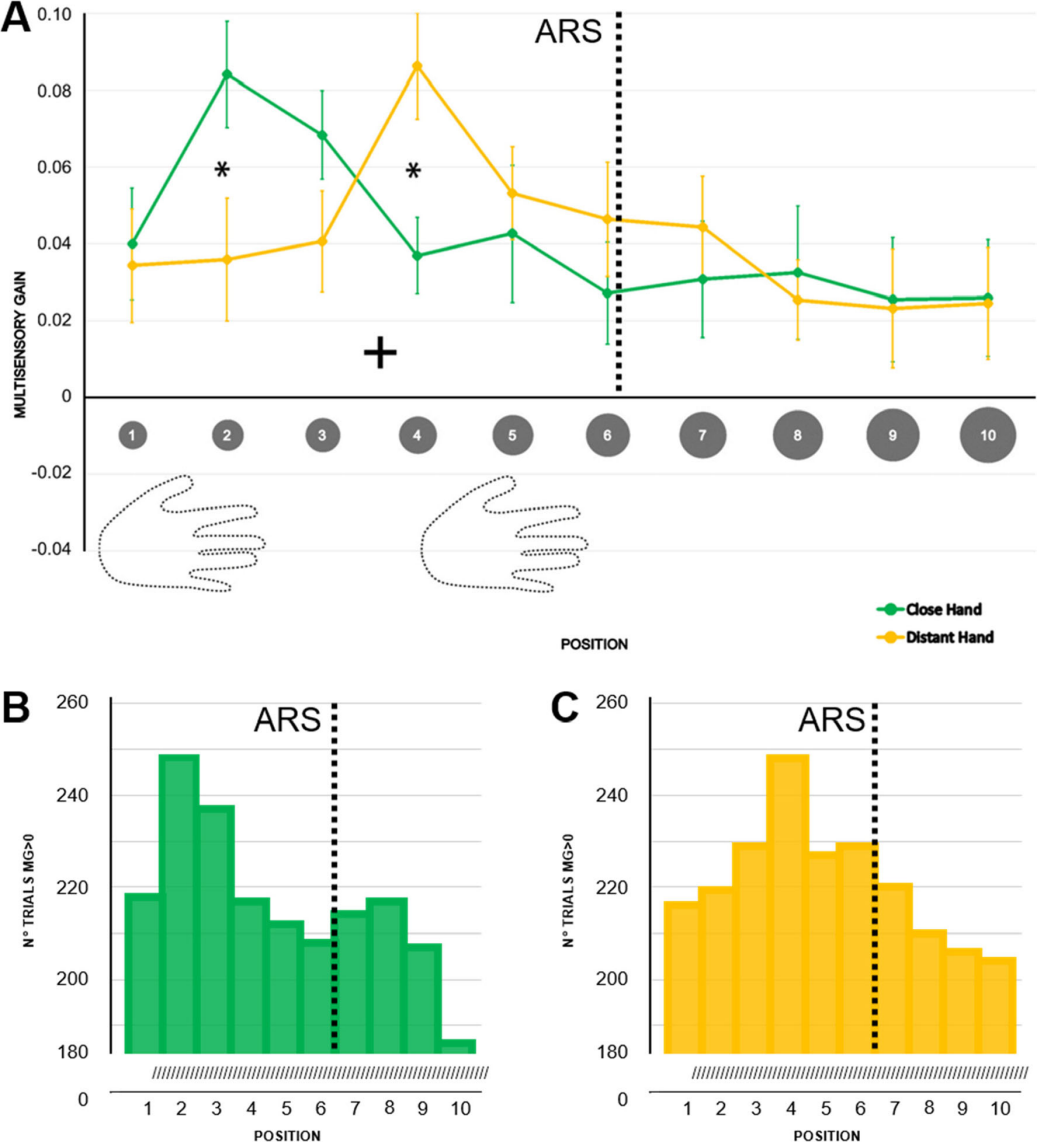
Different patterns of hand-centered multisensory facilitation within ARS. **a** Multisensory gain (MG) values along the ten visual positions, ranging from near to far space, for the distant (yellow) and the close (green) hand conditions. Higher values of MG represent stronger facilitation in terms of RT in the multisensory condition than in the unisensory tactile baseline (by definition, MG = 0). Error bars represent the standard error of the mean. Asterisks represent a significant difference (*p* < 0.05, corrected). **b** and **c** Number of trials reporting MG values greater than zero (unisensory tactile baseline) along the ten visual positions, ranging from near to far space, for the close (**b**) and the distant (**c**) hand conditions

A V-Position*Hand repeated-measures ANOVA (Fig. 2a) revealed a significant main effect of *V-Position* (F_(5.85,128.71)_ = 3.52, *p* = 0.003, η^2^_p_ = 0.14), further modulated by hand position, as indicated by the significant interaction (F_(6.45,141.85)_ = 3.47, *p* = 0.002, η^2^_p_ = 0.14). Tukey-corrected multiple t-test comparisons revealed faster responses in V-P2 than in V-P4 and in all the positions from V-P6 to V-P10 when the hand was close (all p_s_ < 0.05 except V-P2 vs. V-P8, *p* = 0.054); responses were faster in V-P4 than in V-P1, V-P2, V-P3, V-P8, V-P9, and V-P10 when the hand was distant (all p_s_ < 0.05). Critically, the MG was larger in V-P2 when the hand was close than when it was distant (*p* = 0.041). This pattern was reversed in V-P4, where the MG was larger when the hand was distant than when it was close (*p* = 0.022). No other differences were significant.

To identify where multisensory facilitation was significant at the single position level, we ran a series of Bonferroni-corrected t-tests on the MG values versus 0 (i.e., no facilitation). When the hand was close, the MG significantly differed from 0 in V-P2 and V-P3 (all ps < 0.05). In contrast, when the hand was distant, the MG was larger in V-P4, V-P5 (all ps < 0.05) and marginally in V-P6 (*p* = 0.055). Figure 2 shows the number of trials reporting MG values greater than 0 with the hand close (2b) and distant (2c). The density peak shifted coherently with the position of the hand within ARS. Similar results were obtained by analyzing the delta RT for both the ANOVA and the t-tests (see Appendix – Experiment A1). Furthermore, the results of Experiment S1 show that this multisensory facilitation does not depend on sheer attentional factors.

These findings highlight the hand-centered nature of the multisensory facilitation, occurring in different locations, depending on hand position. From this, one would expect (1) the facilitation to be maximal in correspondence with hand location and to decay with distance from it and (2) the bell-shaped pattern of facilitation to follow the hand when it changes position. To test the first prediction, we modelled our data to a Gaussian curve. To test the alternative hypothesis, namely that facilitation spreads all over the ARS to decay when approaching the reachable limit, we compared the Gaussian to a sigmoid function fitting (Canzoneri et al., 2012; Serino et al., 2015). The sigmoidal curve could fit the data for a limited number of participants (distant hand: 5/23 subjects, 21.7%; close hand: 9/23 subjects, 39.1%). Instead, fitting the Gaussian curve to the same data accommodated convergence problems for a higher number of participants (distant hand: 14/23 subjects, 59.9%; close hand: 15/23 subjects, 65.2%).

**Fig. 3:**
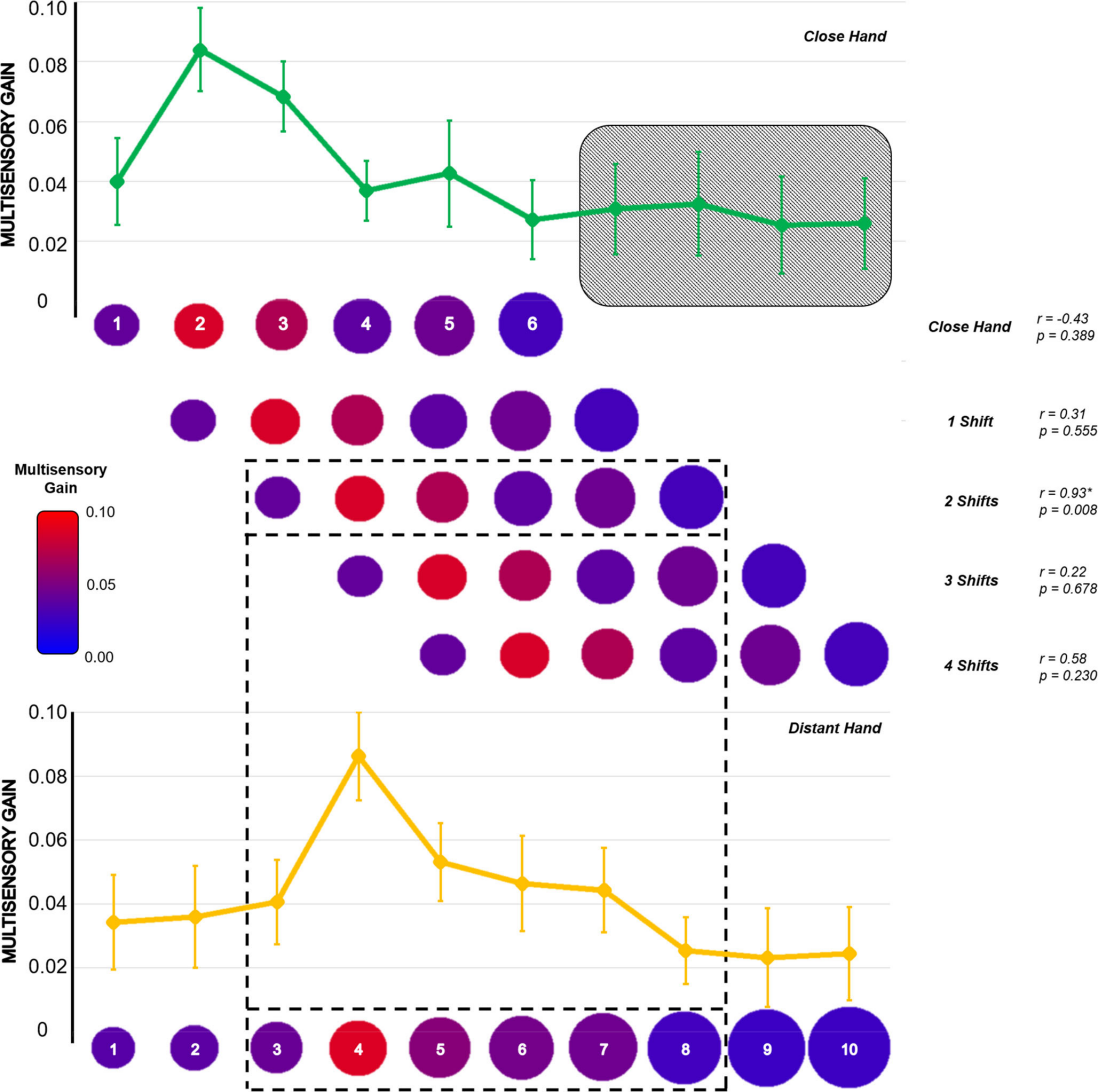
The spatial pattern of MG shifts and follows the hand within reaching space. Cross-correlation analysis of distally shifting the pattern of MG values for all reachable positions with the hand close. Red colors represent higher MG values. Values of Pearson’s r and p values are reported for all the correlations performed. The black grid highlights the only significant correlation (*p* < 0.05)

The second prediction, that the bell-shaped facilitation should shift following the hand, was confirmed by the estimation of the position of the peak of the Gaussian curve in each hand position: with the hand close, the peak fell between V-P2 and V-P3 (2.34 ± 1.51); with the hand distant, it fell between V-P4 and V-P5 (4.15 ± 1.28). We then performed a cross-correlation analysis testing whether the curves reported for the two hand positions overlapped when considered in absolute terms. We reasoned that shifting the position of the hand – within the ARS – should bring to an isomorphic facilitation around the new hand position. This would imply the maximum correlation between MG values emerge when the close-hand curve shifts distally, towards the distant-hand position curve. We considered the first six values of MG with the close hand (from V-P1 to V-P6, i.e., the reachable positions) and correlated this distribution with six values of the MG for the distant hand (Fig. 3). We found the maximum correlation (r = 0.94 *p* = 0.005) when shifting the close hand distally by two positions. No other correlations were significant (all p_s_ > 0.20).

### Discussion

The results of Experiment 1 clearly indicate that PPS and ARS are not superimposable. Yet we cannot exclude that a reachability judgment task might still capture some of the PPS features. To investigate this possibility, we performed three experiments adopting this task and the same settings of Experiment 1. The results of Experiments S2 and S3 (Appendix) replicated well-established findings about the ARS, including the overestimation of its limit (Bourgeois & Coello, 2012; Carello et al., 1989). However, they failed to show any similarity with PPS, either in terms of absolute extent (ARS is larger than PPS) or in position-dependent modulation (PPS is hand-centered, whereas ARS is not, see Appendix, S2 and S3). To allow a full comparison, in Experiment 2 we made the reachability judgment task as similar as possible to the tactile detection task, using the same hand positions and multisensory stimulations.

## Experiment 2

### Methods

#### Participants

Twenty-five (16 females; mean age = 24.44 years, range = 18–41; mean arm length = 78.46 ± 7.26 cm) participants matching the same criteria as Experiment 1 participated in Experiment 2.

#### Stimuli and apparatus

*Visual* and *tactile* stimuli were identical to those used in Experiment 1.

#### Design and procedure

We took advantage of an ARS multisensory task by asking participants to perform reachability judgments while tactile stimuli were concurrently presented with the visual stimulus. Experiment 2 was meant to assess whether the multisensory stimulation (in addition to having the hand visible and in the same positions as Experiment 1) could either induce hand-centered facilitation in the reachability task performance and/or change the extent of the reachability limit. We employed the same settings as in Experiment 1 and applied the same tactile stimulation to the right index finger, placed in either the close or the distant position. However, in this case the tactile stimulus was task-irrelevant. Overall, 160 randomized V and 160 randomized VT trials were presented for each hand position, administered in two blocks in a randomized order. The order of hand positions was counterbalanced across participants.

#### Analyses

Similar to Experiment 1, we tested the classifier on the MG patterns and performed the same procedures already described on delta RTs and MG. The percentage of “reachable” responses per position was calculated and then fitted to sigmoidal and normal curves, as in Experiment 1. We fitted the curves separating hand positions and type of stimulation (unimodal visual vs. multisensory visuo-tactile). *Hand* (close vs. distant)*Stimulation (visual vs. visuo-tactile)*Model (Gaussian vs. Sigmoid) ANOVA on RMSE (root mean square error) values assessed which model best fitted the data, both at the individual and at the group level. Either way, the best-fitting model for these data was the sigmoidal curve. Thus, we investigated the PSE and slope values by subjecting them to two separate repeated-measure ANOVAs with *Hand* (close vs. distant) and *Stimulation* (visual vs. visuo-tactile) as within-subject factors.

### Results

Participants were accurate (>90% hits, <2% false alarms). We computed for each subject two vectors of MG values, as in Experiment 1, and we could leverage a similar data-driven classifier to discriminate the close from the distant hand. Prediction accuracy was lower than in Experiment 1 (0.36, 18/50 correct classifications) and not significantly higher than chance level (one-tailed binomial test *p* = 0.98), indicating that the classifier failed to distinguish between hand positions within ARS.

Moreover, the V-Position*Hand within-subject ANOVA on the MG did not reveal any significant effect (Hand: F_(1,24)_ = 0.83, *p* = 0.37; V-Position: F_(6.31,151.56)_ = 1.20, *p* = 0.31; Hand*V-Position: F_(5.35,128.5)_ = 1.82, *p* = 0.11). However, the significant intercept (F_(1,24)_ = 9.80, *p* = 0.005) confirmed the general facilitation produced by multisensory stimulation, with respect to the unisensory one. Multiple Bonferroni-corrected comparisons revealed that none of the positions presented an MG significantly different from 0 (all p_s_ > 0.05) when the hand was close. V-P5 and V-P6 differed from 0 (all p_s_ < 0.05) when the hand was distant. Similar results were obtained by analyzing the delta RT, both with ANOVA and with t-test (see Appendix – Experiment 2).

**Fig. 4:**
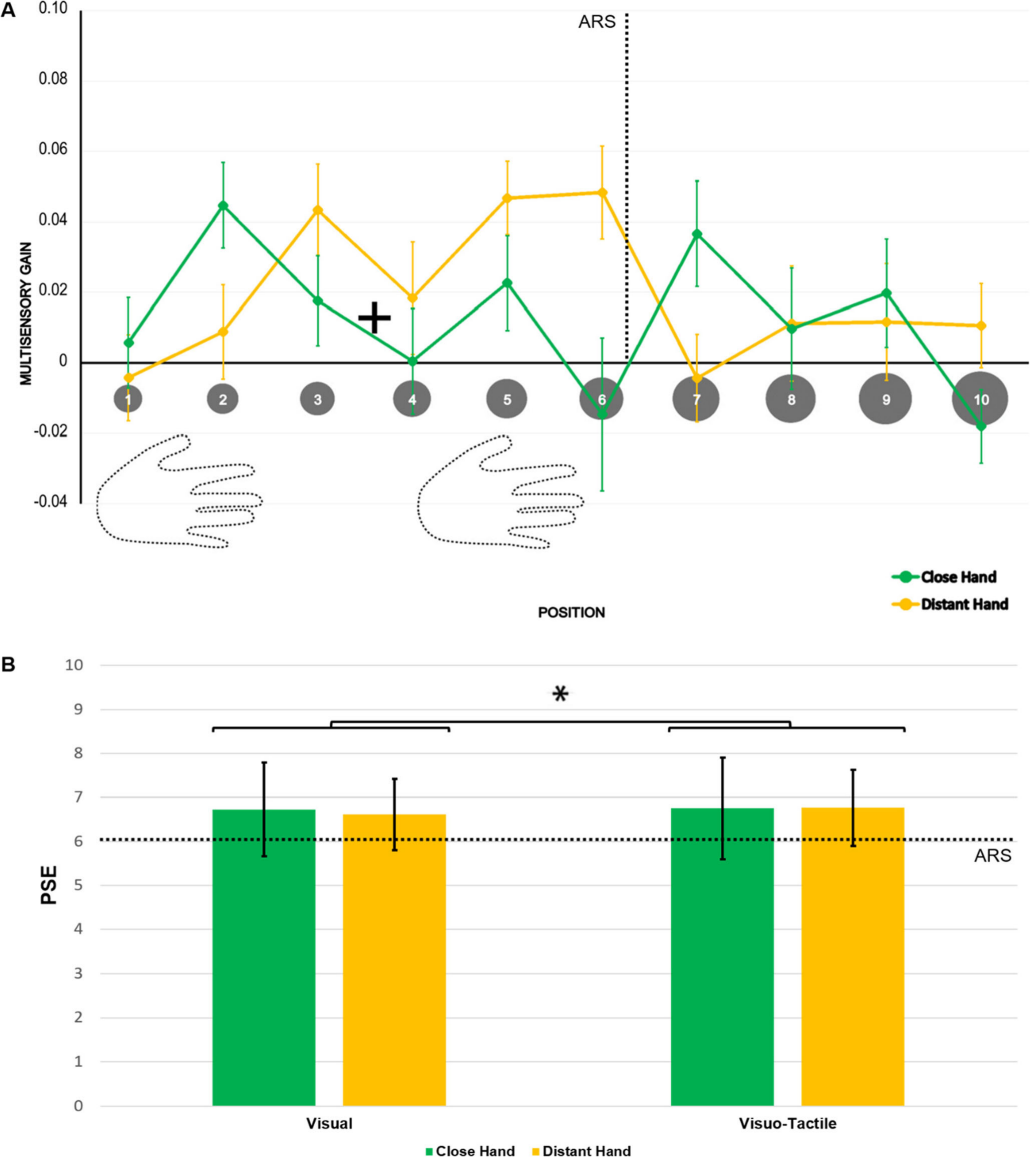
No hand-centered MG spatial patterns in a reachability judgment task. **a** Multisensory gain (MG) values along the ten positions, ranging from near to far space, for the close (green) and distant (yellow) hand conditions. Higher values of MG represent a stronger facilitation in terms of RT with respect to the unimodal visual baseline (by definition, MG = 0). Error bars represent the standard error of the mean. No significant differences between hand postures emerged. **b** PSE values calculated for both unimodal visual and multisensory visuo-tactile conditions for both hands. Error bars represent the standard error of the mean. Asterisks indicate a significant difference between unisensory and multisensory conditions (*p* < 0.05)

Reachability judgments were then fitted to sigmoidal and Gaussian curves. Within-subject ANOVA on the RMSE of these models was performed with a *Model* (sigmoidal vs. Gaussian)*Stimulation (visual vs. visuo-tactile)*Hand (close vs. distant) design. The sigmoidal curve reported the best fit, irrespective of stimulation type and hand position (Model*:* (F_(1,24)_ = 220.11, *p* < 0.001, η^2^_p_ = 0.90)). For each variable, we estimated the coefficients of the sigmoid, obtaining the PSE and the curve slope. Through a *Hand* (close vs. distant)*Stimulation (visual vs. visuo-tactile) within-subject ANOVA on PSE values, we observed a main effect of stimulation type (F_(1,24)_ = 4.38, *p* = 0.05, η^2^_p_ = 0.15): the mean PSE was closer to the body in the unimodal visual (mean ± SE = 6.67 ± 0.17) than in the multisensory visuo-tactile condition (6.76 ± 0.18). The main effect of *Hand* (F_(1,24)_ = 0.07, *p* = 0.79) and its interaction with *Stimulation* (F_(1,24)_ = 3.49, *p* = 0.07) were not significant. Last, we performed a *Hand* (close vs. distant)*Stimulation (visual vs. visuo-tactile) within-subject ANOVA on slope values. Neither main effects (*Hand*: F_(1,24)_ = 1.75, *p* = 0.20; *Stimulation*: F_(1,24)_ = 0.35, *p* = 0.56) nor the interaction (Hand*Stimulation: F_(1,24)_ = 0.27, *p* = 0.61) were significant (Fig. 4).

## Discussion

We contrasted two theoretical views about PPS and ARS: one proposing they are different, the other opposing they are the same. Our findings clearly point against the latter, whether contrasted in terms of their spatial extent, by using their respective gold-standard paradigms and measures, or in terms of pattern of multisensory facilitation.

Due to obvious differences between paradigms, we did not compare the multisensory facilitation directly. We rather reasoned that, would PPS and ARS be the same spatial representation, using their typical paradigms applied to the same body part we should obtain similar results. Our visuo-tactile version of the reachability judgment task confirms previous findings on the extent and overestimation of ARS (Bootsma et al., 1992; Bourgeois & Coello, 2012; Carello et al., 1989; Coello & Iwanow, 2006), but its comparison with the PPS multisensory task resulted in two main advances arguing against the PPS-ARS identity.

First, we observed that multisensory facilitation depends on hand position, peaking in correspondence with its location and deteriorating with distance from it. Notably, this near-hand facilitation effect is independent of attention orienting (see Appendix, S1 results). Thus, PPS is smaller than the ARS, either objectively (from V-P1 to V-P6) or subjectively (PSE) measured. Were they superimposable, we should have observed faster RTs for all the reachable positions of visual stimulation. Both the classifier and the location-specific differences indicate instead that different spatial patterns of multisensory facilitation emerge for the close- and distant-hand positions, despite being both within the ARS limits. Interestingly, we add that overestimation is not modulated by hand vision (see Appendix, Experiments S2 and S3), and is independent of the position of the hand (Experiments 2 and Appendix, S3).

Second, our findings indicate that ARS is not hand-centered, whereas PPS is. In Experiment 2, adapting the reachability judgment task to a multisensory setting, the only significant effect was a general multisensory facilitation, spread over the ten positions tested: there was no modulation as a function of stimulus reachability or hand proximity, which, on the contrary, define PPS (Experiment 1). Therefore, ARS is not encoded in a hand-centered reference frame. Indeed, hand position was robustly classified from the distribution of MG in Experiment 1 (PPS), but not in Experiment 2 (ARS). Thus, the proximity of visual stimuli to the hand – not their reachability – predicts the increase in multisensory facilitation. Cross-correlation and univariate analyses further demonstrated that visual boosting of touch is hand-centered, following changes in hand position. In sum, here we show that (1) PPS does not cover the entire ARS, (2) ARS is not hand-centered, and (3) ARS is not susceptible to multisensory stimulation. Taken together, these results combine to show that PPS and ARS are not superimposable. Previous neuroimaging (Brozzoli et al., 2011, 2012) and behavioral studies (di Pellegrino et al., 1997; Farnè et al., 2005; Serino et al., 2015) reported body part-centered multisensory facilitation within PPS. Here we disclose that the facilitation is isomorphically “anchored” to the hand: present in close positions when the hand is close, it shifts to farther positions when the hand is distant, without changing its “shape.” Notably, the facilitation pattern fits well a Gaussian curve, similar to what is observed in non-human primate studies (Graziano et al., 1997) and in line with the idea of PPS as a « field », gradually deteriorating around the hand (Bufacchi & Iannetti, 2018).

The amount of multisensory facilitation observed in Experiment 1 for the position closest to the trunk (V-P1, thus clearly within ARS) is also remarkable. First, it is lower than that observed in correspondence of the close-hand PPS peak (between V-P2 and V-P3) and, second, it is comparable to that obtained for all the out-of-reach positions (V-P7 to V-P10), irrespective of hand distance.

These findings are consistent with what one would predict from neurophysiological data. Studies on non-human primates requiring reaching movements performed with the upper limb found activations involving M1, PMv and PMd, parietal areas V6A and 5, and the parietal reach region (Buneo et al., 2016; Caminiti et al., 1990; Georgopoulos et al., 1982; Kalaska et al., 1983; Mushiake et al., 1997; Pesaran et al., 2006). In humans, ARS tasks require judging stimulus reachability (Carello et al., 1989; Coello et al., 2008; Coello & Iwanow, 2006; Rochat & Wraga, 1997) or performing reaching movements (Battaglia-Mayer et al., 2000; Caminiti et al., 1990, 1991; Gallivan et al., 2009). Brain activations underlying these tasks encompass M1, PMd, supplementary motor area, posterior parietal cortex, and V6A, as well as the anterior and medial IPS (Lara et al., 2018; Monaco et al., 2011; Pitzalis et al., 2013; see Filimon, 2010, for review). Therefore, despite some overlap in their respective fronto-parietal circuitry, PPS and ARS networks do involve specific and distinct neuroanatomical regions, in keeping with the behavioral differences reported here.

At odds with previous studies employing looming stimuli (Canzoneri et al., 2012; Finisguerra et al., 2015; Noel et al., 2015, b; Serino et al., 2015; but see Noel et al., 2020), we used “static” stimuli flashed with tactile ones to avoid inflating the estimates of multisensory facilitation. Looming stimuli with predictable arrival times induce foreperiod effects that, though not solely responsible for the boosting of touch, may lead to overestimations of the magnitude of the facilitation (Hobeika et al., 2020; Kandula et al., 2017). Most noteworthy, the findings of the attentional control experiment provide the first behavioral evidence that multisensory near-hand effects may be appropriately interpreted within the theoretical framework of peripersonal space coding. This study therefore offers a bias-free (Holmes et al., 2020) protocol for fine-grained mapping of PPS.

In conclusion, this study provides an empirical and theoretical distinction between PPS and ARS. Discrepancies concern both their spatial extent and their behavioral features, and warn against the fallacy of conflating them. A precise assessment of PPS is crucial because several researchers exploit its body part-centered nature as an empirical entrance to the study of the bodily self (Blanke et al., 2015; Makin et al., 2008; Noel et al., 2015, b). Moreover, our results have direct implications for the study of interpersonal space, defined as the space that people maintain with others during social interactions. Several studies drew conclusions about interpersonal space using reachability tasks (Bogdanova et al., n.d.; Cartaud et al., 2018; Iachini et al., 2014). The present findings make clear that using these tasks does not warrant any conclusion extending to PPS, or informing about its relationship with the interpersonal space. Instead, they highlight the need to investigate the potential interactions between PPS and ARS, as to better tune rehabilitative protocols or brain machine interface algorithms for the sensorimotor control of prosthetic arms, for which multisensory integration appears crucial (Makin et al., 2007; Suminski et al., 2009, 2010).

## Supplementary Information

ESM 1 (DOCX 1324 kb)
