# Supplementary material for: Peripersonal and reaching space differ: Evidence from their spatial extent and multisensory facilitation pattern
Source: Psychon Bull Rev. 2021 Jun 22;28(6):1894–905. doi: 10.3758/s13423-021-01942-9 (PMC8642341; doi:10.3758/s13423-021-01942-9)
Supplement: Supplementary file 1 — (DOCX 1324 kb) [file 13423_2021_1942_MOESM1_ESM.docx]

Appendix

Experiment I

We calculated the *delta RT,* obtained as the difference between the mean tactile RTs (baseline) for the specific condition (close or distant hand) and visuo-tactile RTs. Averaging the results per condition, we obtained a delta value for each visual position, from V-P1 to V-P10, for each hand posture. Larger values of *delta RT* indicate faster responses. We thus compared these deltas to zero (i.e., the absence of multisensory facilitation) correcting the multiple tests for Bonferroni correction. These comparisons highlighted a significant facilitation from V-P4 to V-P6 (all p_s_ < 0.05) when the hand was placed in a distant position, with a marginally significant difference in V-P7 (p = 0.06), whereas moving the hand to the close position resulted in a significant facilitation only in V-P2 and V-P3 (all ps < 0.05). Subsequently, we performed a *Hand* (close vs. distant) * *Position* (V-P1 to V-P10) within-subject ANOVA on these delta values to compare the multisensory facilitation obtained at each hand position and to obtain a measure of the extent of this facilitation (namely, the extent of the hand-centred PPS, see Figure S1). Moreover, between-hand position comparisons allowed us to observe whether the position and the extent of this PPS-related facilitation moved in a hand-centred fashion. The main effect of Hand was not significant (F_(1,22)_ = 0.01, p = 0.94), whereas the main effect of Position was significant (F_(5.86,129.02)_ = 3.50, p = 0.003, η^2^_p_ = 0.14). However, this effect was modulated by hand location (significant Hand*Position interaction, F_(6.60,145.23)_ = 3.39, p = 0.003, η^2^_p_ = 0.13). Tukey-corrected multiple comparisons within position revealed significantly faster responses in V-P2 versus V-P4, V-P6, V-P7, V-P9 and V-P10 for the close hand (all p_s_ < 0.05), with a marginally significant difference between V-P2 and V-P8 (p = 0.060); for the distant hand, V-P4 resulted in significantly faster responses than V-P1, V-P2, V-P3, V-P8, V-P9 and V-P10 (all p_s_ < 0.05). Comparing the hand positions, we observed greater facilitation in V-P2 with the close hand than with the distant hand (p = 0.053) and the opposite pattern was observed in V-P4 (p = 0.037). No other differences were significant. Due to the limited number of participants who reported a good fit with the sigmoidal curve on MG values, it was not possible to directly compare the RMSE values obtained with those concerning the fitting of a Gaussian curve. We thus performed a chi-square test with Yates’ continuity correction (Yates, 1934) on the percentages of best fit cases for the distant hand (21.7% for the sigmoidal curve, 59.9% for the Gaussian one), revealing that the Gaussian model fit for a larger proportion of subjects than the sigmoid model (χ^2^_(1)_ = 5.739, unilateral p = 0.008). The same was true with the hand in the close position (39.1% for the sigmoidal curve, 65.2% for the Gaussian one), though the difference was marginally significant (χ^2^_(1)_ = 2.178, unilateral p = 0.069). This may be due to the number of visual positions available in the space preceding the peak of facilitation with the hand close. Indeed, with the hand placed in the distant location, several visual positions before and after the peak of facilitation were present, providing optimal conditions for testing both Gaussian and sigmoidal models. By contrast, with the hand placed close, only one stimulus position was available before the peak, providing equally optimal conditions for the sigmoidal model as the hand distant condition but non-optimal conditions for the Gaussian model. It is possible that this configuration of the setting influenced the fit by biasing sigmoidal over Gaussian fitting in the condition with the hand close. Despite this, however, the Gaussian curve still resulted as the best fitting for most participants.


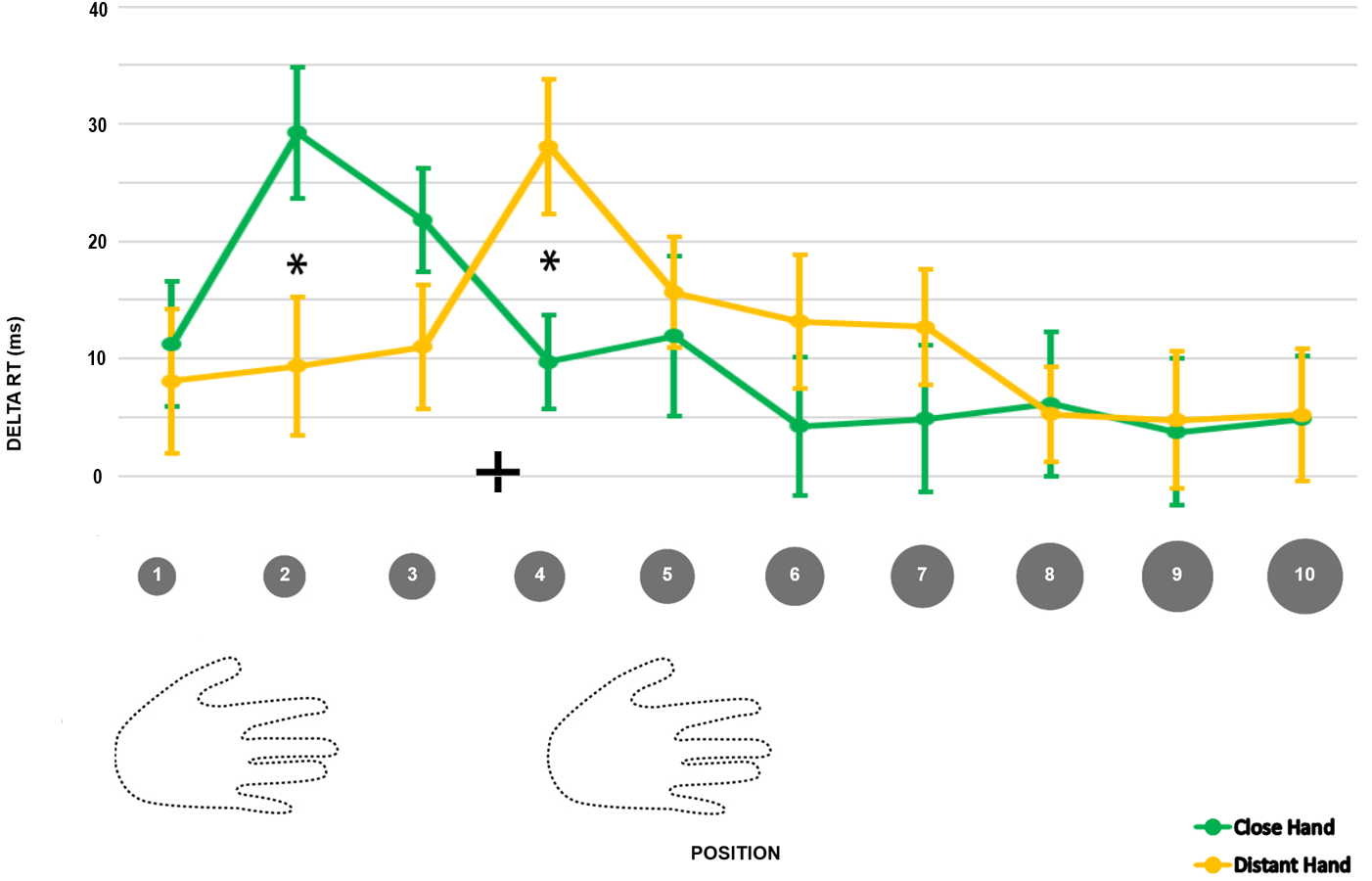


**Figure S1.** Delta RT values along the 10 visual positions, ranging from near to far space, for the distant (yellow) and the close (green) hand conditions. Higher values of delta RT represent a stronger facilitation in terms of RT for the multisensory visuo-tactile condition than for the unisensory tactile baseline (by definition, delta RT = 0). Error bars represent the standard error of the mean. Asterisks represent a significant difference (p < 0.05, corrected).

Experiment II

We calculated the delta RT as done in Experiment I. Thus, we compared the averaged delta for each position to 0 (namely, the absence of facilitation). Bonferroni-corrected multiple comparisons reported only a marginally significant facilitation in V-P2 in the close hand condition (p = 0.058), whereas significant facilitations emerged in V-P3, V-P5 and V-P6 with the distant hand (all p_s_ < 0.05). This was the first main difference from Experiment I, in which a facilitation emerged with both hand positions.

We subjected these values to a *Hand* (close vs. distant) * *Position* (V-P1 to V-P10) within-subject ANOVA to compare the facilitation obtained in the same visual positions with different hand locations. Neither the main effect of Hand (F_(1,24)_ = 0.56, p = 0.46) nor the effect of Position (F_(6.56,157.38)_ = 1.17, p = 0.33) were significant. In the same way, the Hand*Position interaction was non-significant (F_(5.42,130.19)_ = 2.01, p = 0.08). Therefore, Experiment II only highlighted a general effect of the multisensory stimulation over the unimodal visual stimulation, as reported by the significant intercept (F_(1,24)_ = 14.91, p < 0.001); however, this effect was independent of the position of the visual stimulation and was not hand-centred. These results are reported in Figure S2.


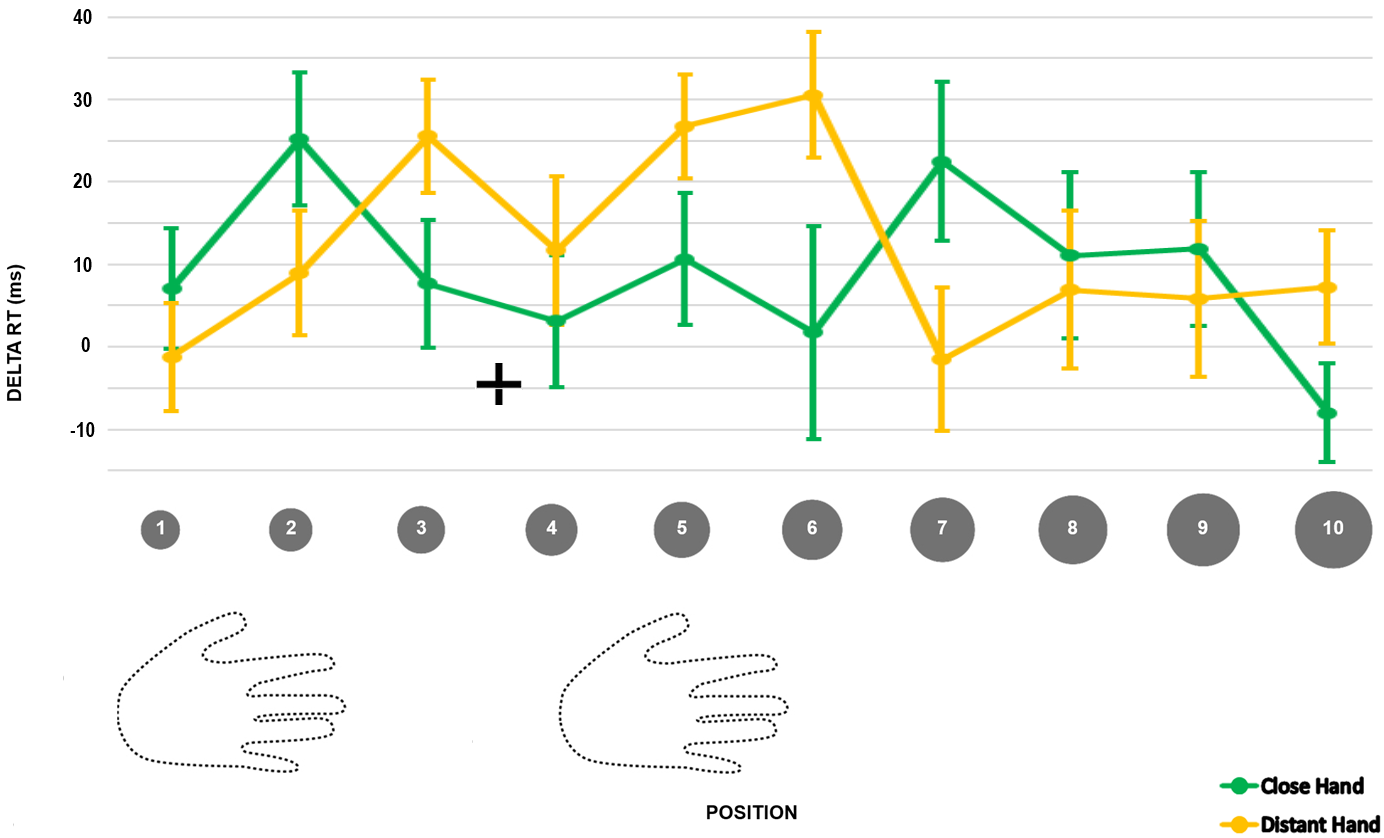


**Figure S2.** Delta RT values along the 10 visual positions, ranging from near to far space, for the distant (yellow) and the close (green) hand conditions. Higher values of delta RT represent a stronger facilitation in terms of RT for the multisensory visuo-tactile condition than for the unisensory tactile baseline (by definition, delta RT = 0). Error bars represent the standard error of the mean.

Starting from the raw reachability judgments, we calculated for each participant the percentage of "reachable" responses per position of visual stimulation, separating the type of stimulus (visual or visual-tactile) and the hand position (close or distant). We then subjected these percentages to an ANOVA 2 (Hand: close or distant) * 2 (Condition: V or VT) * 10 (Visual position, from V-P1 to V-P10). We observed that the main effect of Position is significant: F_(1.53,36.69)_ = 331.63, p < .001, η^2^_p_ = 0.93. The post-hoc with Bonferroni correction show a clear difference between the judgments relating to the first five positions (not statistically different between them, all p_s_ > 0.05) and those relating to the last five (all p_s_ > 0.05). Since the position V-P6 is the objective limit of participants’ reachable space, these results therefore seem to confirm that the subjective judgment becomes less secure at this limit. Moreover, the interaction Hand*Condition is significant: F_(1,24)_ = 7.13, p = 0.013, η^2^_p_ = 0.23. Bonferroni -corrected post-hocs show a difference between the reachable response rate of the unisensory visual condition (mean ± se = 61.1 ± 1.65) and the visual-tactile condition (62.8 ± 1.59), but only when the hand is in the far position (p = 0.004). Even if the absence of a significant interaction with the visual stimulation position prevents us from verifying it directly, we believe it is legitimate to hypothesize that this difference can be related to that found in terms of PSE in the modeling analyses of these judgments. A PSE located at a greater distance in the visual-tactile condition, in fact, involves a greater percentage of "reachable" responses in this condition in the positions closest to the objective limit of reaching. No other significant effect emerged. The percentage of reachability judgments is reported in Figure S3.


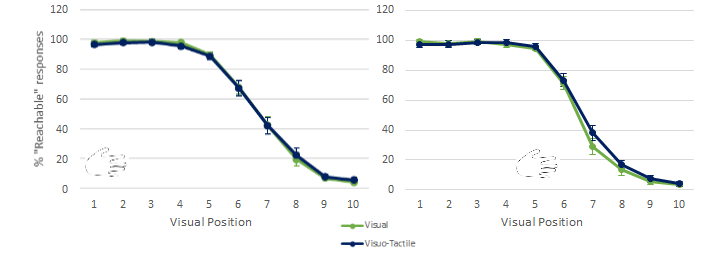


***Figure S3.*** Percentages of reachability judgments for the unisensory visual (green) and multisensory visuo-tactile (blue) conditions along the 10 positions of visual stimulation for the close (left panel) and distant hand (right panel). The 6^th^ visual position represents the objective reachable limit. Error bars represent the standard error of the mean.

Experiment S1

The hand-centered multisensory facilitation described in Experiment I could contain some contribution of attention: it is possible that a greater attentional focus towards a specific position of visual stimulation could influence performance, accelerating the response. Although this possibility has already been ruled out in the literature (Makin et al., 2009), we ran the Experiment S1 in order to test for the presence of hand-centered facilitation also in case when the attentional focus is shifted away from the hand. As illustrated in Figure S4A-B, we asked our participants to place their right hand on the table, in the same position adopted in Experiment I for the near-hand condition, and to perform a go/no go tactile detection task. Tactile and visual stimuli were identical to those of Experiment I, but in this case we took advantage of only two positions of visual stimulation, thereafter “V-Close” (corresponding to V-P2 of Experiment I) and “V-Distant” (corresponding to V-P5 + 3 cm), equidistant from the fixation cross (15 cm closer and farther respectively). In this way, we were able to exploit the position of visual stimulation that most of all facilitated the performance with the close hand in Experiment I and we opposed a far visual stimulation at the same distance from the position of the gaze and within the reaching space. The diameter of the visual stimulations was corrected for retinal size as a function of their distance, established according to the length of the participant's right arm, as in Experiment I. We then projected a circular ring (original diameter 3 cm, corrected for retinal size, duration 50 ms) 8 cm leftward to the fixation cross 150 ms before the tactile stimulus. This ring could be completely closed or show a small opening in one of four possible positions (randomized order). We asked our participants to keep their eyes fixed on the fixation cross and to respond as quickly as possible to tactile stimulation, but only if the ring in the left hemispace was closed. Therefore, to ensure good performance, subjects had to shift their attention to the left, while the target and visual stimuli were presented on the right. Being at the same distance from the fixation cross and the ring, neither of the two visual stimuli could be attentively favored over the other.

We established our sample size through an a priori power analysis (G*Power 3.1.9.2), hypothesizing a power of 0.85 and α = 0.05. We thus performed a paired t-test and a Pearson’s correlation between the subject-averaged multisensory gain in V-P2 and V-P5 in the close-hand condition of Experiment I to obtain the effect size (d = 0.71) and the correlation between measures (r = 0.65). Consequently, we needed to recruit at least 20 participants in our Experiment S3 (N = 20, 13 females; mean age = 27.9, range = 22-53; mean arm length = 78.3 cm).

According to Experiment I, each participant was presented with 16 repetitions for each condition (unisensory tactile, close visuo-tactile and distant visuo-tactile) with a "go" signal. To these were added 8 trials per condition with the "no go" signal and 20% of catch trials (visual unisensory stimulation or no stimulation), equally divided between "go" and "no go" signals.

Participants were globally accurate (mean accuracy on “go” trials > 95%, with no difference between the three conditions: F_(1.94, 36.84)_ = 0.76 p = 0.47) and paid attention to the visual stimulus projected on the left (only 6 responses in “no go” trials, 1.25%). To be consistent with Experiment I, only the participants with a mean accuracy greater than 70% (average accuracy of the conditions of Experiment I considered) were considered for subsequent analyses (no participant excluded). RTs greater than 2.33 SD from the participant’s mean in each condition were filtered out as outliers (2.6%). We thus calculated the delta RT as already described in Experiment I and we performed a paired t test between subject-averaged delta values for the Close and the Distant visual conditions. As reported in figure S4C, the difference was significant: t_(19)_ = 2.35, p = 0.030, Cohen’s d = 0.54, indicating faster responses to touches when the irrelevant visual stimulus was presented close to the hand (mean ± se = 22.58 ± 4.02 ms) compared to far from it (4.22 ± 4.96 ms). Coherently with the formula illustrated in Experiment I, we calculated the multisensory gain per participant and per condition, comparing the facilitation obtained in the two visual conditions through a paired t test. As for the delta RT, the difference was significant: t_(19)_ = 2.34, p = 0.031, Cohen’s d = 0.54. In line with our hypothesis, in fact, the facilitation of the performance linked to multisensory stimulation is greater when the visual stimulus is presented near (mean ± se = 0.035 ± 0.008) rather than far from the hand (0.005 ± 0.008).

These results therefore demonstrate that the effects observed in Experiment I cannot be due only to attentional factors, as they persist even after a shift of attention to a point equidistant from the stimulations used and far from the hand.


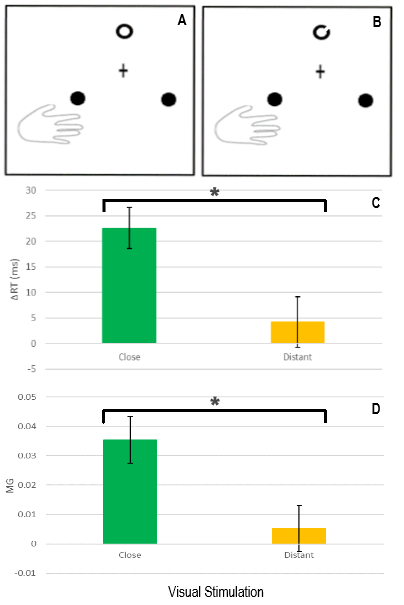


*Figure S4.* *Ruling out attentional factors. A-B) Positions of right hand, fixation cross and visual stimuli Visual stimuli (here displayed as black circles) were projected one at a time, in one of the two possible positions (Close or Distant), corrected for retinal size. Tactile and visual stimuli were presented alone (unisensory) or coupled synchronously with each other (multisensory). Leftward to the fixation cross, a close (A) or open (B) ring represented the “go” and “no go” conditions, respectively.*

*C) Delta RT values for the Close (green) and Distant (yellow) visual stimulations. Higher values of delta RT represent a stronger facilitation in terms of RT for the multisensory visuo-tactile condition than for the unisensory tactile baseline (by definition, delta RT = 0). Error bars represent the standard error of the mean. Asterisks represent a significant difference (p<0.05).*

*D) Multisensory gain (MG) values for the Close (green) and Distant (yellow) visual stimulations. Higher values of MG represent a stronger facilitation in terms of RT for the multisensory visuo-tactile condition than for the unisensory tactile baseline (by definition, MG = 0). Error bars represent the standard error of the mean. Asterisks represent a significant difference (p<0.05).*

Experiment S2

Experiment S2 aimed to extract participants’ (N = 25, 13 females; mean age = 26.17, range: 20-33; mean arm length = 79.37 ± 6.07 cm) reachable limit by asking them to judge the reachability of the 2D visual stimuli adopted in Experiment I.

Procedures were identical to those of Experiment II, with the following exceptions. Participants could not see their hands, kept on their laps under the table. No tactile stimulation was delivered. They were asked to respond (reachable or not reachable) as fast as possible by pressing the left or the right foot pedal (order counterbalanced across subjects). A single block of 160 randomized trials was administered, including 16 visual (V) stimulations per position.

We computed the percentage of ‘reachable’ answers per position and fitted them to sigmoidal and normal curves, as in Experiment II. Through a paired t-test, we compared the root mean square error (RMSE) resulting from the fitting of a sigmoidal and a normal curve (both with 2 parameters) to these judgements. To test for possible differences in the temporal domain between the judgements of reachable and non-reachable positions, we analyzed participants’ RTs (Bourgeois & Coello, 2012) for each of the 10 positions by subjecting them to repeated measures ANOVA with Position (V-P1 to V-P10) as a within-subject factor.

Accuracy of the performance was globally high (> 99%). Comparing the RMSE resulting from the fitting of a sigmoidal and a normal curve to the judgements we obtained a significant difference (t_(24)_ = - 4.269, p < 0.001): residuals were smaller for the sigmoidal curve, indicating it provided a better fit for the data. When assessing the estimated coefficients of this curve, we obtained two parameters: the PSE (mean ± SE = 6.23 ± 0.15) and the slope (mean ± SE = -3.22 ± 0.55; see Figure S5).

As for Experiment 2, the percentages of "reachable" judgments were subjected to an ANOVA with Position (from V-P1 to V-P10) as within-subject factor. We observed a significant effect (F_(2.1,48.3)_ = 275.59, p < .001, η^2^_p_ = 0.92) that we further investigated though Tukey-corrected post-hoc. Again, the first five visual positions (not statistically different between themselves, all p_s_ > 0.05) reported significantly higher percentages of “reachable” responses compared to the last five (all p_s_ < 0.001). These results are in line with the objective limit of reachability of our participants (V-P6) and are represented in Figure S6.

The repeated measures ANOVA on RTs with Position (V-P1 to V-P10) as a factor did not report a significant effect: F_(1.25, 28.7)_ = 2.13, p = 0.152. Moreover, no differences between the RTs of judgement of reachable and unreachable positions emerged, preventing this variable from discriminating between stimuli within and beyond reach.

Experiment S3

A main difference between Experiments I, II and S2 was the vision of the hand. Therefore, we ran Experiment S3 (N = 24, 14 females; mean age = 23.75, range: 18-38; mean arm length = 79.28 ± 5.10 cm) with the aim of introducing into our reaching task the same modulation of the hand we adopted in Experiments I and II. Previous studies adopting reachability judgement tasks to evaluate PPS extensions, in fact, required participants to place the hand in a visible position (Bourgeois & Coello, 2012). Therefore, we ran Experiment S3 identical to Experiment S2, but having the hand visible and in the same positions as in Experiment I. RTs and percentage of answers were computed the same way, but the close and distant hand conditions were run in separate blocks (160 randomized trials per block with the order counterbalanced).

As above in Experiment S2, sigmoidal and normal curves (both with 2 parameters) were separately fitted on the percentages of judgements, as a function of hand position. RTs were subjected to *Hand* (close vs. distant) * *Position* (V-P1 to V-P10) within-subject ANOVA.

Accuracy was high (>99%) and no differences between conditions emerged. In this case, the normal curve did not converge for 3 participants in the distant hand condition and for 2 participants in the close hand condition, whereas the sigmoidal curve provided a convergent fitting for all participants for both hand positions. Paired t-tests on the RMSE revealed a significant difference between the sigmoidal and the normal curve fitting, both for the distant (t_(20)_ = -5.32, p < 0.001) and for the close hand (t_(21)_ = -6.09, p < 0.001) positions: in particular, the sigmoidal curve reported the best fitting, as shown by smaller residuals, independent of the position of the hand. We then calculated the estimated coefficients of the sigmoid for each hand position, obtaining the PSE and the slope of the curve. Through a paired t-test, we compared the PSE for the distant (mean ± SE = 6.36 ± 0.15) and close (mean ± SE = 6.13 ± 0.22) positions; the difference was not significant (t_(23)_ = 1.446, p = 0.162), showing that the subjective judgement of the reachable boundary did not change depending on the hand location. We obtained the same result comparing the slope of the curves: t_(23)_ = -1.770, p = 0.090 (distant hand mean ± SE = -3.71 ± 0.65, close hand mean ± SE = -2.40 ± 0.44, see Figure S4). To study the contribution of the vision of the hand, we compared through an unpaired t-test the PSE and the slope values obtained in Experiment S2 (right hand not visible, placed on the right leg) with those obtained with the close hand in Experiment S3. The differences were not significant, both for the PSE (t_(40.24)_ = -0.405, p = 0.688) and for the slope (t_(45.39)_ = 1.17, p = 0.250).

**Figure S5: Subjective limit of reachability.** a) PSE and b) slope values obtained in Experiment S2 (No Hand) and S3 (Distant or Close Hand). Error bars represent the standard error of the measure.


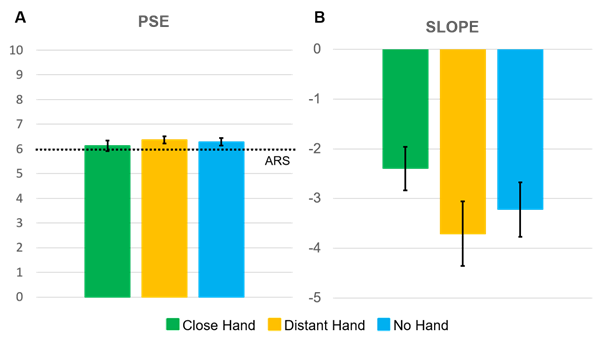


As done for Experiments II and S2, we performed a within-subject ANOVA 2 (Hand, Close vs Far) * 10 (Visual Position) on the percentages of "reachable" judgments issued by our participants. We observed a significant effect of Position (F_(2.06,47.33)_ = 268.62, p < .001, η^2^_p_ = 0.92) and a significant Hand * Position interaction (F_(4.14,95.14)_ = 3.43, p = .011, η^2^_p_ = 0.13). This interaction is significant due to a higher percentage of reachable responses in V-P5 and V-P6 (all p_s_ < 0.05, Bonferroni corrected) when the hand is distant. This could underline the fact that it is easier for participants to discriminate the reachability of a stimulation in border positions when the hand is close to that limit. These results are reported in Figure S6.


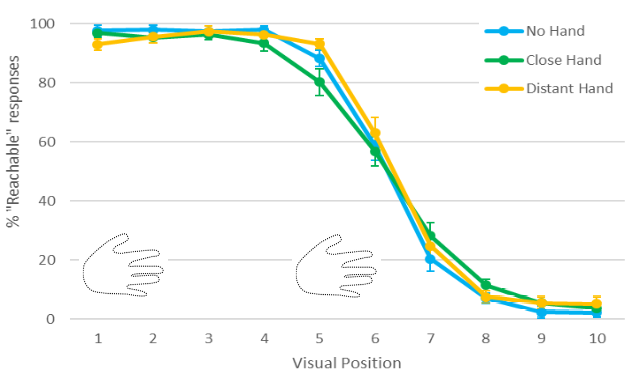


***Figure S6.*** *Percentages of reachability judgments along the 10 positions of visual stimulation for the close (green, Experiment S3), distant (yellow, Experiment S3) and no hand conditions (light blue, Experiment S2). The 6^th^ visual position represents the objective reachable limit. Error bars represent the standard error of the mean.*

RTs were subjected to *Hand* (close vs. distant) * *Position* (V-P1 to V-P10) within-subject ANOVA. None of the main effects was significant: Hand (F_(1,23)_ = 0.67, p = 0.423) or Position (F_(1.40,32.27)_ = 1.23, p = 0.293). Similarly, the Hand*Position interaction was not significant (F_(4.75,109.16)_ = 0.68, p = 0.629). These results highlight the fact that reachability judgements over visual stimuli are not modulated by the position of the hand (Experiment S3) or by its visibility (comparing Experiment S2 with Experiment S3). This corroborates our hypothesis regarding the difference between PPS and ARS: contrary to what was observed in Experiment I, the position of the hand does not modulate the judgement speed.
